# Decoding enhancer complexity with machine learning and high-throughput discovery

**DOI:** 10.1186/s13059-023-02955-4

**Published:** 2023-05-12

**Authors:** Gabrielle D. Smith, Wan Hern Ching, Paola Cornejo-Páramo, Emily S. Wong

**Affiliations:** 1 Victor Chang Cardiac Research Institute, 405 Liverpool Street, Darlinghurst, NSW Australia; 2 School of Biotechnology and Biomolecular Sciences, UNSW Sydney, Kensington, NSW Australia

## Abstract

Enhancers are genomic DNA elements controlling spatiotemporal gene expression. Their flexible organization and functional redundancies make deciphering their sequence-function relationships challenging. This article provides an overview of the current understanding of enhancer organization and evolution, with an emphasis on factors that influence these relationships. Technological advancements, particularly in machine learning and synthetic biology, are discussed in light of how they provide new ways to understand this complexity. Exciting opportunities lie ahead as we continue to unravel the intricacies of enhancer function.

**Supplementary Information:**

The online version contains supplementary material available at 10.1186/s13059-023-02955-4.

## Introduction

Enhancers are a class of genomic *cis*-regulatory elements that play crucial roles in controlling gene expression [1–3]. The term ‘enhancer’ was first coined in 1981 to describe an element in the simian virus 40 (SV40) genome that enhanced beta-globin gene expression in HeLa cells by 200-fold [4]. We now know that enhancers function in shaping organismal phenotype across all life stages by instructing context-specific transcriptional profiles that vary with cell type, tissue, organ, life stage, and environment [5–7]. Most enhancers reside in non-protein coding regions; however, exonic enhancers have also been shown to drive tissue-specific expression patterns [8].

**Fig. 1:**
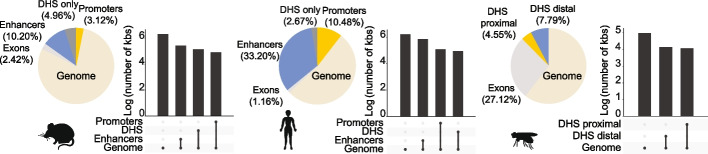
Proportion of *cis*-regulatory elements in animal genomes. Percentage of the mouse, human, and fruit fly genomes occupied by putative *cis*-regulatory elements based on histone marks, accessible chromatin (from DNase I hypersensitive sites (DHS)), and protein-coding regions (exons; those overlapping predicted regulatory elements are excluded). Upset plots show the log_10_ number of kbs for each region. Mouse accessible regions were profiled in 55 cell and tissue types and *cis*-regulatory elements were defined by the analysis of H3K4me1, H3K4me3, and H3K27ac histone marks across multiple tissues [15]. Human *cis*-regulatory elements were defined based on 18-state ChromHMM chromatin models across 98 epigenomes [16, 17]; these elements were defined based on their combination of histone modification profiles across the genome. Enhancer and promoter regions were defined as the union of multiple states (EnhWk, EnhA1, EnhG1, EnhBiv, EnhA2 and EnhG2 for enhancers; TssBiv, TssFlnk, TssA, TssFlnkD and TssFlnkU for promoters). Fruit fly accessible regions were profiled in five embryonic stages (S5, S9, S10, S11, and S14) [13], where DHS regions were separated into proximal (± 1 kb from TSSs) and distal regions

A significant proportion of mammalian genomes, between 11 and 33%, has been classed as potential enhancers based on genomic associations with markers of enhancer activity across cell and tissue types. This “enhancer real estate” is significantly larger than the 2% of the genome that comprises of protein-coding genes [9–15] (Fig. 1). However, our understanding of the transcription-driving activity of enhancers across cellular contexts remains limited. The vast majority of candidate enhancers have not been validated based on their ability to drive transcription. Large-scale validation approaches have been mainly restricted to in vitro applications and a handful of cell types.

In recent years, machine learning models trained on epigenomics data has shown remarkable utility in predicting enhancers and their transcription factor (TF) binding sites, including those genetic variants that impact chromatin accessibility [18–24]. For instance, deep learning models have been used to predict the influence of genetic mutations in melanoma by scoring variants that affect chromatin accessibility in melanoma cell states [25, 26]. ﻿Computational methods combined with high-throughput synthetic biology have allowed the testing of fully engineered sequences for enhancer activity, broadening our understanding of enhancer evolution and developmental control [27, 28].

Notably, enhancers have been also identified in plants [29] using techniques such as massively parallel reporter assay (MPRA) [30] and chromatin accessibility maps [31, 32]. Plant and animal enhancers appear to have different properties. For example, poised and active animal enhancers are often marked by H3K4me1, but this does not seem the case in plants (reviewed in [33]). This is an important area; however, our focus here will be on metazoan enhancers as they have been most extensively studied.

In this review, we provide an overview of enhancer structure, organization, and mechanisms of action, emphasizing the challenges posed by their rapid evolution and robustness that make classification based on DNA sequences alone difficult [7]. We discuss the use of high throughput, data-rich approaches, particularly in unraveling the “enhancer code” and anticipate that current advancements in molecular biology and computer science will deepen our understanding of sequence-specific enhancer activity, leading to new insights into regulatory mechanisms and evolution. This knowledge will also be essential for incorporating machine learning models in formal disease diagnosis [34, 35].

## Mechanisms of action

Enhancers are classically thought to exert their regulatory effects via physical interaction, whereby looping chromatin, supported by structural proteins, brings enhancers, and their associated transcription factors (TFs) into physical proximity with the target promoter [36]. This can bypass linear distances which can span up to a megabase [37–40]. However, the transcription of enhancers, known as eRNAS [41–44], has also raised questions that the eRNA itself could serve to regulating looping or by forming chromatin domains either locally or in *trans* [45–47].

**Fig. 2:**
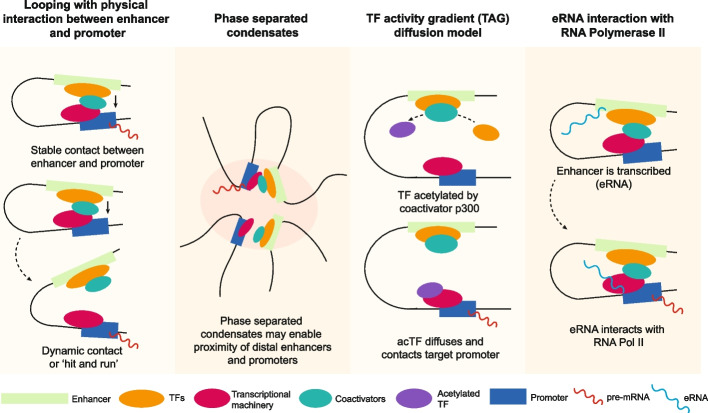
Mechanisms of enhancer action. Enhancer action on target promoters can occur via looping that enables physical contact in either a stable or dynamic manner in agreement with the evidence of proximity from chromatin conformation experiments and DNA FISH [39]. Alternative mechanism of action include the phase separation of enhancers, promoters, and associated proteins into condensates which can provide the proximity required for enhancer-promoter contact without looping [49, 50]; the diffusion of factors from enhancers to promoters, such as acetylated TFs [51], forming a chemical gradient that enables specific targeting of promoters within close proximity without direct enhancer-promoter contact; and a role for eRNAs by triggering pause release of RNA Pol II at target promoters [54]

Enhancer-promoter looping is associated with topologically associating domain-facilitated contact, compatible protein profiles, and distance requirements between the enhancer and target promoter [38, 48]. These features are not universal and other enhancer-promoter communication mechanisms without looping have been described (Fig. 2). Phase separated condensates, a mechanism of biochemical compartmentalization, may enable the action of super enhancers given parallels between the molecular cooperativity in cellular body formation and the assembly of regulatory factors at high density during super enhancer activation [49, 50]. A hypothetical model of communication via diffusion, described as TF activity gradients (TAG), has been proposed to regulate enhancer-promoter communication via short-distance diffusion of acetylated TFs [51]. This model eliminates the need for physical contact between enhancer and promoter and could provide an explanation for observations of proximity, but not contact, between some active enhancers and promoters [52, 53]. Another possible mode of enhancer action could involve the transcription of enhancers into eRNAs, which have been implicated in transcriptional regulation via interaction with NELF, stimulating Pol II pause release and transcriptional elongation eRNAs [54]. Multiple reports of *trans*-acting interactions across homologous chromosomes, a phenomenon known as transvection, has also been characterized in fruit flies [55, 56].

## Enhancer identification

Enhancers can be identified through various methods, including conservation analysis, genome-wide correlation with chromatin data, measuring eRNAs or transcription of a reporter gene, and using CRISPR-based methods. While sequence conservation can be used to map enhancers from one species to another, many enhancers cannot be identified by sequence conservation alone [5, 7].

The availability of large-scale epigenomics datasets has allowed researchers to analyze genome-wide patterns of regulatory signals to identify enhancers and other genomic elements using correlative approaches. Chromatin enrichment in H3K4me1, H3K27ac, and the chromatin modifier p300 histone acetyltransferase are considered genome-wide markers of regions with enhancer activity [57, 58]. Since enhancers need to accommodate TFs and the associated cofactors necessary for their activation, they are nucleosome deficient. Accessible chromatin away from transcriptional start sites (TSSs), inferred by DNase-seq and ATAC-seq, are also used to detect candidate enhancers [59, 60].

Enhancer RNAs (eRNAs) are bidirectionally transcribed from TSSs within enhancers and tend to overlap known enhancer histone marks. eRNAs can provide higher specificity in enhancer detection compared to histone modifications due to the single base resolution of nascent transcript [61, 62]. eRNAs are identified using assays that enrich for active 5’ TSSs, such as CAGE, or nascent transcript assays, such as PRO-seq and GRO-seq, where the expression level of transcripts is considered a functional quantification of enhancer activity [11, 61, 63, 64]. Single-cell transcriptomic profiling can capture the 5′ end of transcripts (CAGE) to identify enhancers at single cell resolution [65]. However, transcription is not exclusive to enhancers but is also a feature of promoters suggesting the regulatory roles of enhancers and promoters are more interchangeable than once thought [62, 66]. Bidirectionally transcribed promoters can act as strong enhancers, while enhancers can also act as weak promoters [63, 67]. These signals provide insights into the role of enhancer as transcriptional hubs and has raised intriguing questions into the biological roles of eRNAs [68]. Beyond the idea that eRNAs are mere passengers of TF activity, some eRNAs have been shown to have specific functions [43], including regulation of spatial organization associated with the production of lncRNA [69], and the formation of transcriptional condensate through m6A methylation of nascent RNAs [70]. Notably, despite significant overlap between the sets of enhancer candidates identified by different approaches, there are incongruencies between the different methods of enhancer annotation [71]. Based on these genome-wide approaches, millions of enhancer candidates have been identified across tissues and cell types in metazoans. However, the validation of these candidates is a significant bottleneck.

In vivo transgenic approaches are used to validate enhancers in a developmental context providing critical spatiotemporal information across the different cell types of a developing animal. These experiments involve the transgenesis of a cassette containing a test sequence with a minimal promoter and a reporter, which may be randomly integrated into the genome or targeted to a safe harbor/neutral landing site using CRISPR/Cas9 [35, 72]. A dual-fluorescence, dual-CRE transgenic cassette can also be used to measure the activities of normal human enhancers and the same enhancer encoding a putative disease variant simultaneously in F1 zebrafish [73]. However, in vivo transgenesis using a reporter gene is low throughput in vertebrates and tend to lack endogenous context. High throughput validation of enhancer activity, including MPRA and perturbation-based methods will be discussed in a following section.

## Enhancer sequence code

**Fig. 3:**
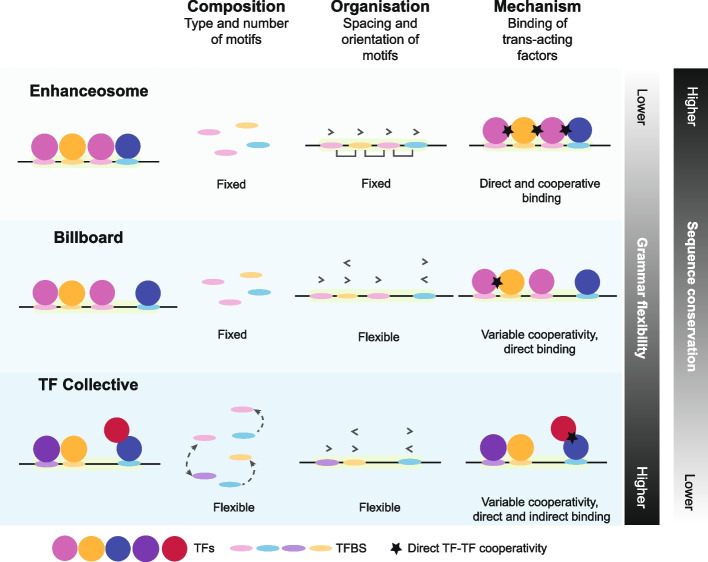
Current models of enhancer grammar. The flexibility of the type, number, orientation, and spacing of motifs within an enhancer sequence can vary (reviewed in [5, 78]). The enhanceosome model relies on fixed composition, number, and organization of motifs that support direct and cooperative binding of TFs [80], corresponding to enhancers with low sequence flexibility and thus increased sequence conservation. A greater degree of sequence flexibility is found in the billboard [79] and TF collective [76] enhancer models which incorporate variable motif organization and TF cooperativity

Mechanistically, enhancers are considered as clusters of TF binding sites (TFBS) that recruit *trans*-acting factors and target protein-coding gene promoters [74–77]. Parameters including the type, arrangement, and orientation of binding motifs, collectively referred to as enhancer ‘grammar’ implying a common syntax or logic to the enhancer region, can also play a role in determining enhancer activity (reviewed in [5, 78]). Several models of enhancer organization have been proposed, including the billboard [79], enhanceosome [80], and TF collective models [76]. Each model varies in the mode of DNA-binding protein occupancies and organizational structure (Fig. 3). The enhanceosome model requires the strict arrangement of TF binding sites and direct TF cooperation, while the billboard and TF collective models describe a more flexible arrangement of binding with indirect cooperation—the latter featuring an increased role for protein-protein interactions (reviewed in [2, 5, 78]). Enhancers are thought to fall along a spectrum of these models and the precise mechanisms by which they function can vary depending on the specific enhancer and the cellular context. As such, motif arrangements, mutations or deletions can have varying degrees of impact depending on the enhancer [81, 82]. For example, in the arrangement of ZicL and ETS binding sites that drive gene expression patterns key to *Ciona* notochord development, suboptimal spacing can be tolerated if compensated by stronger TF-DNA binding affinities [83].

An emerging view, supported by studies using transgenic activity assays in vitro and in vivo, suggests that for most enhancers, grammar may be relatively weak and TF occupancy is often sufficient to confer enhancer function [27, 81, 84–87]. It is also important to note that although the major attention is on TF binding motifs, nearby sequences can also impact TF binding by altering DNA shape, chromatin accessibility and allosteric regulation [88–91]. Epigenetic modifications, such as DNA methylation, also interplay with TF binding [92]. While DNA methylation can repress TF binding, some important developmental TFs appear to prefer methylated CpG binding sites [92].

## Challenges to understanding enhancer code: evolution

Enhancers, in comparison to promoters, tend to more evolve rapidly and often without strong sequence constraint [7, 93–96]. Positive selection has been observed in a subset of rapidly evolving human enhancers associated with immune function and development [97, 98]. While some enhancers are highly conserved among vertebrates [99–101], around 50% of candidate enhancers detected in 20 placental mammals are lineage-specific and recently evolved [93]. This dynamic reflects a rapid rate of TF binding site turnover and has been linked to transposable elements [102], which make up a significant portion of mammalian genomes [103–105]. Yet, less than half of the lineage-specific enhancers overlap with transposable elements, suggesting that most new enhancers have originated from non-regulatory sequences that already exist in ancestral genomes [93, 103]. In these cases, non-regulatory sequences may have acquired activity through point mutations that create new TFBS [94, 106–110].

Across animal phyla, conserved enhancer sequences are rare. Of five thousand of candidate enhancers from the sea anemone, *Nematostella*, none shared recognizable sequence similarity to *Drosophila* or zebrafish [111]. Only one example of strict sequence conservation extending beyond bilaterians has been reported among animal enhancers [112]. However, around 10% of human-zebrafish syntenic loci, ~ 300, showed conserved TF binding motif arrangements at regulatory regions [113]. Arrangements of TF binding sites have been used to identify many pairs of putative homologous regulatory elements at conserved syntenic loci that otherwise bear little sequence similarity between human and zebrafish genomes [113].

While detecting conserved enhancers across distant metazoan is highly challenging, genome analyses have identified hundreds of examples of microsynteny (pairs of conserved syntenic genes) across metazoans [114, 115]. The long-term linkage of microsyntenic genes across animal evolution is attributed to the presence of a *cis*-regulatory element within a gene regulatory block (GRB) that controls the expression of a developmental gene (the “target” gene) [116, 117]. GRBs with conserved enhancers are found within topologically associating domains that form regulatory, self-interacting chromatin architectural features facilitating long-range enhancer-promoter contacts [118]. In the case of the *Islet-Scaper* microsyntenic region, a sea sponge *Islet* enhancer was able to drive similar GFP expression patterns to those of endogenous zebrafish *Islet* expression, despite the lack of primary sequence similarity [85]. Similarly, teleost enhancers without detectable evolutionary conservation can direct human gene expression and vice versa [119]. Hence, evolutionary distant animals share similar TFs, TFBSs, and developmental gene regulatory pathways, and enhancer-promoter connections [111, 120–123].

Not all enhancers evolve quickly; some enhancers have stretches of identical sequence that are shared between human, rat, and mouse, and are referred to as “ultraconserved” [99–101]. Ultraconserved elements are often found in large, gene-sparse regions and may represent a subset of a larger group of enhancers that generally have higher levels of sequence conservation and may have substantial differences in their phenotypic contributions [101, 124]. They appear to be characterized by the high occupancy of many TF binding sites [125], which may contribute to their pleiotropy in functional activity between cell types and stages of development, thereby increasing evolutionary sequence constraints [1, 126]. Despite high sequence conservation, mutagenesis at many of these regions does not lead to embryonic lethality, which suggests that these sequences may have negative impacts on fitness at life stages beyond development or are conserved for other unknown reasons [127].

Enhancer conservation also varies between different developmental stages and in different tissue types, although the reasons for this variation are not fully understood [103]. Enhancers defined by ChIP-seq of p300 and open chromatin regions tend to be particularly well conserved at certain critical times during embryogenesis, called the phylotypic stage, when there are similarities in gene expression and body plan within phyla [96, 128]. Cardiac enhancers during mouse embryonic development tend to evolve with less evolutionary sequence constraint compared to forebrain enhancers [95, 96]. Cell-type specific variation may reflect differences in the essential nature of the enhancers or the robustness of the tissues they regulate. Other factors, such as variations in chromatin organization and DNA replication time, may also contribute to the faster evolution of certain enhancers [129].

In summary, our current sequence alignment paradigms appear largely insensitive to *cis*-regulatory conservation. New computational methods based on neural networks is allowing the prediction of tissue-specific enhancers where nucleotide-level conservation is low but the predicted open chromatin in a tissue of interest is conserved [130, 131]. By constraining functional analysis to sequences conserved across great evolutionary distances, we identify only a small proportion of functional information in genomes suggesting new strategies are required.

## Challenges to understanding enhancer code: robustness

The resilience of phenotypes to changes in enhancer activity is closely tied to the rapid evolution of enhancer sequences. The effectiveness of natural selection for a phenotype is influenced by its robustness, which refers to the ability of the phenotype to maintain stability in the face of genetic perturbations. Robustness is a general feature of complex systems that are evolvable (reviewed in [132]).

**Fig. 4:**
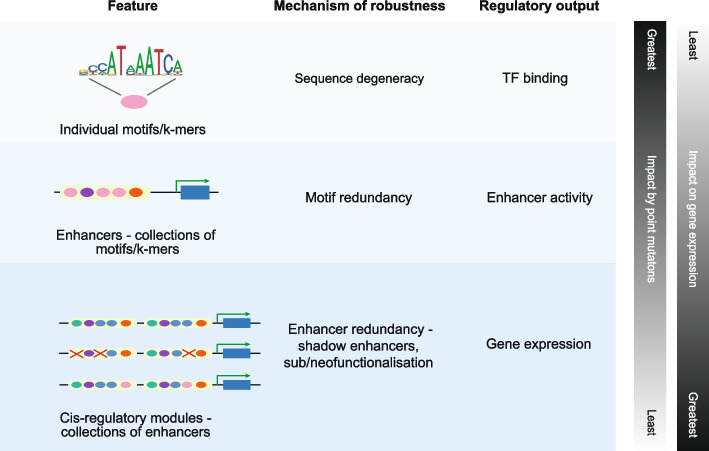
Organizational structures contribute to robustness and sequence divergence. The short length and sequence degeneracy of TF binding motifs, redundancy and flexibility of motif organization within enhancers, and the structure of the *cis*-regulatory module contribute to the overall robustness of *cis*-regulatory elements

Robust enhancers have a high proportion of genetic sequences that do not impact fitness. These “hidden” variants are expected to evolve neutrally. The robustness of enhancers can be attributed to several characteristics at various organization levels: the structure of individual TF binding motifs, the organization of an individual enhancer, and the arrangement of multiple enhancers within a gene regulatory module (Fig. 4). TF binding motifs contribute to robustness by tolerating base substitutions on a position-specific basis, which is called degeneracy. Another approach to maintain stability in gene activity is by having multiple copies of a motif within enhancers and a flexible motif grammar [133–135]. An example of this robustness is found in *Sepsidae* and *Drosophilidae* flies where the relative position and location of key binding sites that drive the eve stripe 2 enhancers have changed, yet the flies show similar stripe 2 expression patterns [87, 136].

The requirement for low affinity TF binding sites (TFBS) for accurate gene expression patterns during animal development can be also viewed as an emergent property of a robust system [137, 138]. In the *Drosophila* Hedgehog morphogen gradient, low-signaling regions are only active with weak TF affinity [139]. Similarly, the *Ciona* developmental enhancer Otx-a has a “suboptimal” motif sequence and motif arrangement [138]. Low affinity binding contributes robustness because weak binding affinity sites are more likely to randomly occur than strong ones. Most randomly generated TFBS are mutationally distant from the highest affinity sequence [140, 141]. Thus, maintaining a low-affinity binding site is easier than a high-affinity one. Suboptimal binding promotes specificity in gene expression and prevents ectopic expression in non-target tissues, which may have been an emergent trait of a robust system.

There are several factors that contribute to robustness at the level of gene regulatory models. These include through enhancer redundancy [142–144], the need for multiple TFs to bind together [137, 138], and the transmission of genetic signals through different layers of regulatory information [145]. These mechanisms can help maintain the accuracy of gene regulatory circuits despite sequence divergence at *cis*-regulatory elements.

Enhancer redundancy, or the use of multiple redundant enhancers (shadow enhancers) to drive the same gene expression pattern, increases transcriptional robustness (reviewed in [144]). Shadow enhancers regulate the expression of the same gene, compensating for environmental or genetic alterations to normal developmental programming [143, 146–150]. Many shadow enhancers are partially functionally redundant, with enough overlapping spatial activity maintaining robust developmental gene expression and buffering the impact of genetic variations [150]. Genes with greater regulatory complexity, including more shadow enhancers, results in more robust in gene expression by comparing *cis*- and *trans*-acting genetic variation in *Drosophila* F1 lines [145].

The binding of multiple TFs functions similar to logic gates, masking the impact of mutations and increasing the accuracy of transcriptional control [137, 138, 151–153]. Propagation of genetic signals through multiple regulatory layers helps to maintain the fidelity of gene expression patterns [145, 154, 155]. Thresholds on transcriptional activation or repression can buffer signal variation.

The interplay between evolvability and robustness is a recurring theme in the study of animal regulatory networks. Robustness can promote diversity, leading to the increased evolvability of phenotypes. The short length of TF binding sites allows new TF binding sites to emerge quickly during evolution [156], enabling even random sequences to acquire *cis*-regulatory activities [28, 108]. For example, it takes 0.5–10 million years to evolve the complexity required for a *cis*-regulatory element involved in anterior-posterior axis specification in *Drosophila* blastoderm, starting from a random genome background [107]. In a study using mutational libraries in *Drosophila* embryos, Galupa et al. showed that while existing developmental enhancers are constrained in cell-type specific function, de novo elements harboring TF motifs can drive developmental gene expression across different cell types [28]. Increased levels of sequence variation at developmental enhancers may have propelled speciation and morphological diversity [97]. An experimental evolution study in *E. coli* show that new mutations can become quickly fixed in the population, even in the absence of selection [157].

The concepts of neofunctionalization and subfunctionalization, proposed by Ohno [158] to explain the fate of duplicated genes and the emergence of new functions, also apply to the evolution of duplicated enhancers. Redundancy of function in shadow enhancers can contribute to new gene regulatory networks [142]. The pace of enhancer turnover and larger number of enhancers suggest that these processes occur more frequently in enhancers than in genes.

The mode of TF binding affinity inheritance can also enhance regulatory evolvability. Unlike gene expression, which is often inherited in a dominant or recessive manner, TF binding occupancy at *cis*-regulatory elements typically follows a co-dominant inheritance pattern. This may allow genetic variants that contribute to regulatory differences to be easily selected for, promoting adaptability in gene regulatory networks [6, 145, 159].

## Investigating enhancer activity by high throughput experimentation

**Fig. 5:**
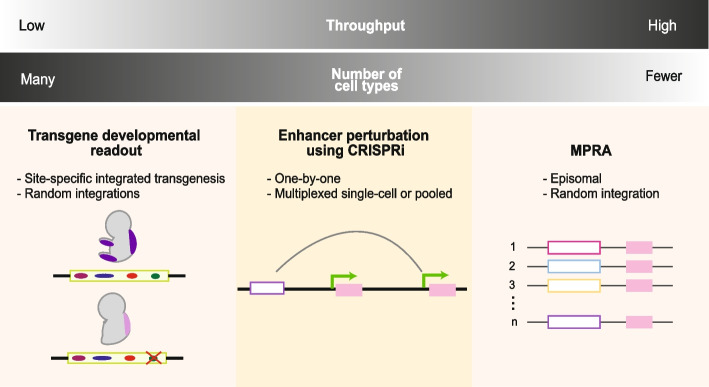
Experimental methods for testing enhancer activity. Methods that are used to assess enhancer activity involve a trade-off between the number of sequences that can be tested and the number of cells assessed at one time. Developmental transgenic approaches can reveal enhancer activity across many cells at the same time on a per sequence basis. On the other hand, massively parallel reporter assays (MPRA) are able to assay thousands of sequences by random integration or in an episomal manner. Perturbation experiments using CRISPR interference (CRISPRi) can reveal transcriptional targets and can be combined with single-cell readouts to increase throughput

Experimental validation of enhancers is necessary to confirm enhancer activity and understand the relationship between enhancer sequence and function. This poses a significant challenge due to the context-specific nature of enhancers and the sheer number of enhancer candidates. Validation of enhancer activity can be performed using transgenic animal models or in high throughput using massively parallel reporter assays (MPRA) and CRISPR-based perturbations (reviewed in [160, 161]) (Fig. 5).

MPRAs employ a library of reporters and high-throughput sequencing to examine potential enhancers [27, 81, 86, 162, 163]. MPRAs can simultaneously assess thousands of potential enhancer sequences, using gene expression as an indicator of enhancer activity [135, 164]. In this approach, a library containing thousands of plasmids, each carrying an enhancer sequence adjacent to a minimal promoter, is introduced into cells or animal models. MPRA libraries may be randomly integrated into the genome allowing the study of chromatin location-specific effects, or remain separate from the genome (episomal), reflecting the overall regulatory capacity in the tested cell type [165–168].

The design of MPRAs, including factors such as oligo length, the relative positioning of the candidate sequence to the promoter, and the choice between integrated or episomal assays, can influence reporter activity. A comparison of nine major strategies by Klein et al. showed that while most MPRA designs correlate well, the location of the enhancer candidate on the plasmid has a more significant impact than the differences between episomal versus integrated assays [169]. Additionally, while sequence orientation generally does not matter, sequence length, which influences the number and type of binding sites present, can strongly influence activity outcomes.

MPRAs have enabled researchers to validate the activity of endogenous *cis*-regulatory elements [135, 170] while facilitating investigations on the impact of human genetic variations [171, 172]. Studies have varied the positioning, orientation, and diversity of TFBS for key pluripotency factors in stem cells revealing that motif grammar is often flexible but mutations within TFBS can disrupt binding and affect activity [135, 164, 172–174].

Limitations to MPRAs include a lack of endogenous chromatin context, and a loss of relevant epigenetic modifications. As MPRAs are typically used in homogenous cell populations, this restricts their application in rare cell types or cells that are challenging to maintain in culture. However, recent advancements have enabled MPRAs to be combined with single cell RNA-seq sequencing, allowing researchers to study enhancers during cell differentiation and paving the way for the evaluation of enhancers in their native cellular contexts [175, 176].

CRISPR-based genetic perturbation screening addresses the limitations of MPRAs by studying enhancers in their natural cellular context. This technique can be applied on a large scale and at single-cell resolution, enabling the investigation of multiple loci by introducing various perturbations to many cells. Activation or repression of regulatory elements can be examined using CRISPR interference (CRISPRi) or CRISPR activation (CRISPRa) or by direct editing of the regulatory sequence. Using pooled guide RNAs and high-content readouts, these methods allow for the determination of direct and indirect relationships between enhancers and genes at multiple *cis*-regulatory elements [177–186]. Although these techniques are usually performed on cells in vitro, there are in vivo applications using adeno-associated viral (AAV) in animal studies [187].

## Using machine learning to dissect *cis*-regulatory elements

Machine learning is transforming our understanding of cis regulatory sequences and their role in gene regulation [188] (Table 1). By using large datasets of multi-omics information, or data from MPRA experiments, deep learning algorithms can identify complex patterns and relationships within the data that may be difficult to otherwise discern. The flexibility of these algorithms has seen them applied to a range of challenging problems. For example, to differentiate between all mapped human *cis*-regulatory elements [18], identify cell-type specific accessible chromatin [20], predict TF binding sites and enhancers across species [189–191], prioritize the impact of regulatory mutations [192], dissect enhancer and promoter grammar [27, 193], and to predict gene expression [163, 194, 195].

**Table 1:** Machine learning models used in the prediction of *cis*-regulatory elements

**Method**	**Core algorithm/architecture**	**Goal**	**Trained model**	**Reference**
Gkm-SVM	Support Vector Machine	To find distinguishing features within regulatory elements	**Class 1**: CTCF ChIP-seq signal enriched regions in GM12878 cell line **Class 2**: Random sequences (matching length, GC and repeat fraction)	[196]
EnhancerFinder	(Multiple Kernel Learning) Support Vector Machine	Enhancer prediction (developmental enhancers)	**Class 1**: Enhancers from VISTA Enhancer Browser **Class 2**: Random regions from genomic background	[197]
RFECS	Random Forest	Enhancer prediction	**Class 1**: p300-binding sites (H1 and IMR90 datasets from NIH Roadmap Epigenome Project) **Class 2**: TSS overlapping DNase-I, and random regions distal to known TSS and p300 sites (H1 and IMR90 datasets from NIH Roadmap Epigenome Project)	[198]
DeepEnhancer	Convolutional Neural Network	Enhancer prediction	**Class 1**: Enhancers from FANTOM5 **Class 2**: Sequences from human reference genome	[199]
DeepSEA	Convolutional Neural Network	To prioritize functional variants at regulatory regions	**Multi-label:** Open chromatin, TF binding and histone mark profiles from ENCODE and Roadmap Epigenomics datasets across multiple human cell types	[192]
DeepBind	Convolutional Neural Network	TF binding prediction	**Class 1**: Protein binding microarrays, ENCODE ChIP-seq peaks, HT-SELEX **Class 2**: Shuffled class 1 sequences (maintaining dinucleotide composition)	[200]
Basset	Convolutional Neural Network	To find distinguishing features within regulatory elements	**Multi-label:** Chromatin accessibility in 164 cell types (ENCODE and Roadmap Epigenomics Consortium)	[20]
DeepSTARR	Convolutional Neural Network	To find distinguishing features within regulatory elements	**Class 1**: Enhancers with developmental activities **Class 2**: Enhancers with housekeeping activities	[193]
BiRen	Convolutional Neural Network + (Gated Recurrent Unit) Bidirectional Recurrent Neural Network	Enhancer prediction	**Class 1**: Human and mouse enhancers from VISTA Enhancer Browser *with* reproducible expression patterns **Class 2**: Human and mouse enhancers from VISTA Enhancer Browser *without* reproducible expression patterns	[22]
DeepMEL	Convolutional Neural Network + (Long-Short Term Memory) Bidirectional Recurrent Neural Network	To find distinguishing features within regulatory elements	**Multi-label:** Melanoma human open chromatin regulatory regions	[26]
DanQ	Convolutional Neural Network + (Long-Short Term Memory) Bidirectional Recurrent Neural Network	To find distinguishing features within regulatory elements; To prioritize functional variants at regulatory regions	**Multi-label:** 919 ChIP-seq and DNase-seq peaks from ENCODE and Roadmap	[201]
AgentBind	Convolutional Neural Network	Predicting TF binding sites	**Class 1**: ENCODE TF binding ChIP-seq data from multiple cell types **Class 2**: Genome-wide excluding Class 1 regions matched for GC content	[202]
ResNets	(Residual Network) Convolutional Neural Network	To find distinguishing features within regulatory elements	**Multi-label:** Enhancer sequences with distinct regulatory architectures (homotypic clusters, heterotypic clusters, enhanceosomes)	[203]
CSI-ANN	Time-Delay Neural Network	Enhancer prediction	**Class 1**: HeLa cell ENCODE data, Human CD4^+^T cell data **Class 2**: Random genomic loci	[204]
EnhancerDBN	Restricted Boltzmann Machine + Deep Belief Network	Enhancer prediction	**Class 1**: Human “positive” enhancers (VISTA Enhancer Browser), DNA methylation, histone marks, GC content **Class 2**: Genomic background matched for length and chromosome distribution to **Class 1**	[205]
BPNet	(Residual Network) Convolutional Neural Network	To predict TF binding profiles at single base-resolution	**Multi-label:** ChIP profiles for TFs	[206]
DNABERT	Bidirectional Encoder Representations from Transformers	To find distinguishing features within regulatory elements	**Multi-label:** k-mers	[207]
Sei	Convolutional Neural Network; linear and non-linear layers with residual connections	Classifies based on > 21,000 types of human chromatin profiles	**Multi-label:** > 21,000 types of publicly available human chromatin profiles (TF binding, histone marks and DNA accessibility) across > 1,300 human cell lines and tissues	[18]
Enformer	Convolutional Neural Network + Transformer	To predict gene expression and chromatin state profile across multiple cell types in human and mouse genomes	**Multi-label:** 5,313 human and 1,643 mouse gene expression and chromatin states at 128 bp resolution from 200 kb of input sequence	[194]
ChromBPnet	Convolutional Neural Network	To predict chromatin accessible profiles at single base-resolution across the genome after removing biases from enzymes used in DNase-seq and ATAC-seq assays	**Multi-label:** SnATAC-seq of human developing cortex	[208]

A general usage example is as follows: a machine learning algorithm is trained on a pre-defined set of features, such as publicly available datasets of functionally validated enhancer sequences, histone markers, and open chromatin, by associating the input data with labels. The algorithm is then able to determine the underlying patterns that contribute to the labeled class. This process, called training, involves minimizing a loss function (e.g., classification error) at each iteration of the algorithm. The training set typically consists of a fraction of the total available dataset, while the test set is a held-out subset used for model evaluation and is not used in training. A diverse training dataset can improve prediction accuracy and reduce bias in the model [197]. Models can also be trained on data from specific biological contexts and then used for inference in different contexts [189–191, 199, 209]. For example, a model trained to distinguish enhancers in one species can be used to infer enhancers in another [189–191]. Training is typically the most time-consuming and memory-intensive part of machine learning and often requires specialized hardware such as GPUs (graphics processing units).

As input, many studies have leveraged large-scale epigenomics datasets from global consortium initiatives, such as the human and mouse ENCODE and NIH Roadmap Epigenomics Consortium projects, which comprise multiple omics readouts across a wide range of cell lines and primary tissues. The Cistrome Data Browser is a useful resource that compiles all publicly available human and mouse ChIP-seq and DNase-seq datasets [210]. The candidate enhancers from primary tissue data have typically not been experimentally tested for enhancer activity. However, some studies have used experimentally validated enhancers, such as the enhancer VISTA database [211], to train machine learning models to identify tissue-specific enhancer syntax [203]. Sequence models trained with activity data from MPRA experiments can be used to identify the sequence basis for regulatory activity [27, 163, 193].

A multitude of machine learning algorithms have now been developed for regulatory element prediction, with neural network frameworks becoming increasingly popular (Table 2). Non-neural network algorithms comprise of a range of machine learning methods, including support vector machines (SVMs), and tree-based approaches such as random forests (RFs) and gradient boosting machines (GBMs) (Table 1).

**Table 2:** Common architectures for *cis*-regulatory classification

**Machine learning algorithm**	**Mechanism**	**Advantages**	**Interpretation**
Support vector machine	Finds a maximal margin hyperplane that best divides data into the required classes	Relatively memory efficient and best suited for high numbers of input dimensions (e.g., k-mers)	With respect to GkmSVN: - Calculation of importance scores at nucleotide resolution using Shapely values, GkmExplain [212] - Introduction of variants in the input sequence and estimation of their impact on the SVM score, deltaSVM [24]
Random forest	Predictions are made from the aggregated result from a set of decision trees, trained in parallel, where each node represents a particular feature	Features are used as explicit classifiers, providing a easy way to interpret the model	- Estimation of feature importance scores, such as Gini score, permutation score, and Shapley values, is a standard practice for dissecting tree ensembles [191, 213] - Partial dependence plots are useful to interpret a random forest; they show the relationship between a given feature and the response variable while other predictor features remain constant [214]
Gradient boosting machine	Uses a series of random forests, and allows for the systematic decrease of a loss function with forests improving on one after another	Yields the benefits of random forests but with added robustness due to having continually improving forests	Similar to random forest
Convolutional neural network (CNN)	Filters of varying sizes slide across the sequence/input unit, capturing patterns and integrating information using cross-correlation to produce a feature map of the sequence	Can learn complex patterns while reducing dimensionality compared to non-convolutional neural networks	Reviewed here [215] - Search for subsequences that activate a convolutional filter and construct PWMs - Attention weights for visualizing feature importance - Propagation of perturbed data through model to observe effects on predictions. This can be done by forward propagation (in silico mutagenesis (ISM)) or backward propagation (e.g., GradCAM, DeepLIFT [216]) - Aggregation of attribution maps to identify globally important sequence motifs (e.g., TFMoDisco [217]) - Initializing filters to known TF motifs (e.g., DanQ [201])
Bidirectional recurrent neural network (RNN) Related: Time-delay neural network	Hidden states in layers preserve information from previous layers, forming a context that contributes to deciding the next action	Captures interdependencies between hidden states	Similar to CNN
Bidirectional Encoder Representations from Transformers (BERT)	Uses an attention-based model used in natural language processing (NLP) tasks	Use self-attention to understand interaction between important regions	- Shapley values can be computed to dissect BERT models [218] - DNABERT-viz was developed to visualize importance scores at nucleotide resolution leveraging self-attention values [207]

Enhancers can be represented as position weighted matrices (PWMs) derived from validated TF binding sites, as k-mers, or using one-hot encoding (reviewed in [7]). One-hot encoding is a method that converts each nucleotide to a numeric variable and commonly used in neural network models. PWMs are easily interpretable but are limited to the motifs of selected proteins. K-mers and gapped k-mers are more flexible representations because they capture all combinations of short sequence patterns, allowing for the de novo discovery of motifs. The gapped k-mer support vector machine (gkmSVM) approach has consistently outperformed its predecessor, kmer-SVM, and has been widely used to analyze enhancer sequences [23, 190, 196]. The most predictive k-mers from these models often match known experimentally confirmed TF binding motifs [21]. The impact of regulatory variants can be assessed by calculating the differences in gkmSVM scores, termed deltaSVM [24]. While gkmSVM is effective and easily interpretable, it may not be able to recognize long-range patterns between motifs due to cooperative or additive TF binding.

Over the past decade, convolutional neural network (CNN) has emerged as a powerful neural network architecture. The complex interconnected multi-layered neuron structure in neural networks allows the algorithm to discern patterns and features that may not be otherwise recognizable [219]. To increase the capacity for such complex pattern recognition, there can be many layers of neurons in these networks, leading to the term “deep learning.” Convolution refers to the use of a filter window of a certain length to smooth out noise while retaining important features.

CNNs can be used alone and as part of hybrid frameworks [219]. Early applications of CNNs to genomic data include CSI-ANN [204], DeepBind [200], DeepSEA [192], and Basset [20] (Table 1). These were trained to predict TF motifs, prioritize functional variants at regulatory regions, and classify features such as chromatin accessibility from the sequence. These methods laid the foundations for other high-performing methods designed for regulatory elements, such as DanQ [201], DeepEnhancer [199], DeepMEL [191], and DeepSTARR [193] (Table 1).

Model performance, measured by the area under the curve comparing false positive versus true positive rates (ROC-AUC), exceeded 80% in many chromatin feature classification tasks, such as distinguishing between cell types. However, this metric may convey an overly optimistic impression of these models’ performance in cell-type classification tasks due to significant class imbalance.

Natural language processing (NLP) models, such as GTP, have achieved impressive capabilities in different tasks and could surpass CNN-only models in detecting distant semantic dependencies within genetic sequences. Large language models may excel at discerning complex dependencies between sequence elements [220] (Table 2). For example, BERT (Bidirectional Encoder Representations from Transformers) [220] has achieved state-of-the-art performance in NLP tasks and holds promise for improving our understanding of the genome. DNABERT, a BERT model pretrained on the human genome using k-mers as inputs, has developed a general-purpose understanding of the genomic semantics and has been applied to classify promoters and identify TFBS [207].

Another transformer-based model, Enformer, is a large deep learning algorithm trained on ~ 7000 human and mouse datasets, that has shown high performance in predicting cell-type accessible chromatin and gene expression across human and mouse genomes [194]. Karollus et al. showed that Enformer has learnt the causal principles of key TFBS at promoters in K562 cells but that it does not sufficiently account for distal enhancer activity [221]. This is likely due to class imbalance as the number of enhancers driving a target gene’s expression decreases with distance away from the gene’s TSS. These findings underscore the importance of conducting further research to determine whether deep learning sequence-based models employ correlative or causal sequence principles in their predictions.

While deep learning algorithms can make accurate predictions, they can also be difficult to interpret and are often referred to as “black box” algorithms due to their lack of transparency [222]. The interpretation of AI models is an area of ongoing development in genomics research (reviewed in [215]). The architecture of a neural network can influence its interpretability, with designs that tend to learn either distributed (partial) or localist (whole) representations of sequence motifs with the latter providing a greater level of insight into network decisions [223]. Several methods have been developed to assign importance scores to individual nucleotides to interpret deep learning models. These include DeepLIFT [224], which uses a difference-from-reference method, and DeepExplainer, which uses Shapley Values [216]. Shapley Value is a concept from game theory that considers the contribution of each feature not just based on its input order but also in all other possible orders, to provide a fair assessment of each feature’s importance. Another method, called TF-MoDISco [217], is specifically designed for motif interpretation and discovery and is able to process sequence importance scores using information from all the neurons of a neural network. This method can also be used with feature attribution importance scores from gapped k-mer support vector machines (GkmExplain) [212]. Clustering algorithms are used in the interpretation of machine learning frameworks to identify important motifs which are then compared to PWMs. The clustering of motifs is key to the interpretation of trained models [191, 217].

Model interpretation can be facilitated by using simple network architectures. For instance, ExplaiNN [225] uses a large series of simple neural networks each of which learns different TF binding profiles making it efficient to train and allowing for global interpretability while sacrificing the ability to capture interactions between different motifs.

Other approaches to interpreting decision-making processes in neural networks include modifying the input data to test the importance of specific nucleotides and analyzing the network structure (for a detailed review, see [180]). Studies have shown that up to 50% of motifs learnt using different machine learning methods do not match any known canonical TFBS. This may be due to algorithmic limitations or that these motifs may have biological roles other than protein recognition.

## Challenges and opportunities

The field of machine learning is rapidly evolving, with models demonstrating great potential in their ability to identify enhancer sequences. Sequence models have the potential to play important roles in prioritizing disease-causing variants and in defining cell-type resolved *cis*-regulatory elements when combined with MPRA and single cell genomics (e.g., congenital heart disease [226]). Despite these exciting developments, there are significant challenges to overcome.

First, deep learning algorithms require a large number of examples in order to learn complex patterns and make accurate predictions, which can be a challenge in the field of genomics where data, especially from validated enhancers, is limited. Because the sequence syntax and logic within enhancers are complex and context dependent, understanding the regulatory code that determines when and where genes are expressed in animals requires access to a large amount of data in diverse cell types and time points. There is a paucity of large datasets and enhancers with validated activity in humans tend to be restricted to a handful of cell lines (i.e., K562) and a subset of evolutionarily conserved enhancers between human and mouse [211].

The use of large-scale datasets, including those generated through consortium initiatives like ENCODE and the NIH Roadmap Epigenomics Consortium, will continue to be a valuable resource for machine learning approaches. Developments in high-throughput molecular validation methods to allow for more cell types to be tested will improve data availability for machine learning models [170, 175, 176].

Second, understanding the specific biological features that drive model predictions and the decision-making process is an area of active research [215]. The development of more accurate and interpretable machine learning approaches can lead to a greater understanding of the complexities of enhancer function and the identification of new regulatory elements and mechanisms. NLP models may generally improve interpretability. Another exciting area of research is latent text-to-image generative models that are being applied to design novel cell-type specific regulatory elements, which when combined with molecular validation can help further elucidate cell-type specific regulatory codes [227].

Proteomics can be used to validate promising findings to gain novel biological insights. The integration of machine learning with experimental validation will be key to fully realizing the potential of these approaches to decipher the intricate relationship between enhancer sequences and activity.

Third, while deep learning algorithms can be highly accurate, they do not always generalize well to new datasets. To be able to accurately transfer knowledge across cell types in different species would be a valuable tool. The use of transfer learning, which involves pre-training a model on a large dataset and then fine-tuning it on a smaller dataset may improve the performance of deep learning models for predicting TF binding sites [209].

Finally, while the development of predictive models that can identify and predict the activity of endogenous and synthetic *cis*-regulatory elements provides an important framework for understanding enhancers, a unified definition of context-specific enhancer activity based on interpretable sequence rules would serve as a basic organizational principle of the regulatory genome.

## Conclusions

A major goal in genetics is to elucidate enhancer sequences to better understand how the genome encodes cell and organismal traits. Enhancers are characterized by features that make them highly flexible and evolvable, including redundancy, modularity, sequence degeneracy, and binding suboptimality. These features provide robustness, but they also make it challenging to decipher the underlying principles of enhancer function.

In silico methods, such as machine learning, combined with single-cell approaches offer new avenues to study enhancers and understand the relationship between their sequence and activity in different in vivo contexts, including rare and transient cell states. While these methods have been successful in identifying candidate enhancers and their gene networks, we are still in the early stages of developing biologically meaningful sequence models that can accurately predict enhancer activity in specific cell types and at specific time points.

Ongoing developments in technology and data collection, including in areas such as single cell genomics, will be critical for advancing our understanding of enhancers and other *cis*-regulatory elements. By leveraging these advances, we can build predictive and interpretable frameworks for understanding the sequence basis of enhancers to gain insights into their role in shaping organismal phenotypes.

## Supplementary Information

**Additional file 1.** Review history.
