# Supplementary material for: Decoding enhancer complexity with machine learning and high-throughput discovery
Source: Genome Biol. 2023 May 12;24:116. doi: 10.1186/s13059-023-02955-4 (PMC10176946; doi:10.1186/s13059-023-02955-4)
Supplement: Supplementary file 1 — Additional file 1. Review history. [file 13059_2023_2955_MOESM1_ESM.docx]

Review History

**First round of review**

**Reviewer 1**

This manuscript provides a review of much recent work on the prediction, validation, and evolution of enhancer sequences and their activity. There's a lot of ground to cover under this scope, and the review touches on most of the relevant literature; however, given the length, it is necessarily somewhat high-level.

I feel that this review would be useful for somone new to the field to help orient them to main themes and approaches. However, I do not think it provides sufficient synthesis, novel perspective, or provocative argument to be of much interest to experts. This is ok if an introduction was the goal, but I prefer reviews that have something for both the novice and expert. I would minimally suggest adding more concrete description of areas in need of future work, beyond the general "machine learning" and "high-throughput assays." What specifically within these areas are the opportunities? What are the challenges of integrating them and validating models?

Here are my detailed comments and suggestions (page.line):

2.3 Typo: enhancers'

2.6 This statement is unclear. Does this describe evolutionary sequence conservation or conservation of activity? I also feel that using the word "exception" suggests that enhancers are very rarely conserved, which is not true.

2.24 This is incorrect. Enhancers can also occur in coding regions. See for example:
https://genome.cshlp.org/content/22/6/1059

2.30 The first paragraph of the Intro is very light on references. Perhaps citing a few relevant earlier reviews could help fill in the gaps.

2.41 I appreciated the detailed discussion of all the features that enhancers sometimes exhibit in this paragraph. However, I think it would be helpful to clarify at the start of the paragraph that these are neither all necessary or sufficient for enhancer activity. This become clear by the end of the paragraph, but was a bit confusing at the start.

3.2 Can you clarify what exactly activity means here. How is it defined? Be a bit more explicit about the distinction being made.

3.24-28 While agree that a sequence-only approach would be great, it isn't totally clear to me that this is possible. Perhaps it isn't all specified in the sequence.

3.59 While it is interesting to mention transvection, I found its mention right at the start of the section on mechanisms without further comment a bit jarring. Perhaps discussing later after discussing the physical interaction model would be more clear.

4.10 "These features may not be universal" I agree! This could be the theme of studying enhancers!

5.1 Typo: constraint

5.6 About the introduction of the different enhancer "grammar" models, I agree that this is necessary for context on the field. However, I think it would be helpful to be clear that these are not universal and there is unlikely to be a single answer. Enhancers likely exist along a spectrum between these extremes.

5.30 I don't think "strategies" is the right word, it implies agency to the enhancers.

5.40 Does this mean confirmed functional activity conservation? There are many functionally (and sequence) conserved human-zebrafish enhancers that probably haven't been tested in more diverged animals. I wonder how many of these at least have evidence of sequence conservation across other animals?

6.35 In this paragraph, I was confused about how the discussion of somatic variation related to differences in germline variation. Can this be clarified?

7.35 I find the definition and discussion of robustness to be unclear. For example, why would this necessarily imply that variants evovle neutrally. For example, shadow enhancers are more conserved than expected. I suggest clearly defining the meaning of robustness in this discussion at the start. I also suggest citing and referring to this recent review on enhancer robustness:
https://www.nature.com/articles/s41576-020-00311-x

7.46 Can this estimate of the time of emergence be explained? What is the starting point?

10.8 Typo: provide

10.17/55 What is meant by "diseased"?

11.2 I feel that more discussion is needed about the lack of agreement/replication and other challenges in interpreting the results of different MPRAs. Reference 153 gets at this, but this challenge isn't described in the text.

11.26 It would be helpful to clarify from the start that these ML models are often not trained on validated enhancers but regions with signatures associated with enhancers.

11.42 This paragraph is lacking citations throughout. Cite relevant reviews or primary lit.

11.48 I disagree with the characterization of ML models into neural network and non-neural network. The non-neural network methods are quite diverse and distinct.

13.46 I'd suggest also citing the Enformer method, since it also predicts many markers of enhancer activity:
https://www.nature.com/articles/s41592-021-01252-x

13.50 Reference for this?

Figure 1: Given the dependence of these estimates on ChromHMM, it would be helpful to explain this method.

Figure 1: Also, how are these fractions sensitive to the very different numberes of cellular contexts included?

Figure 1: Given the debates surrounding the ENCODE-style definitions of function, it would be worth discussing this debate slightly and mentioning conservation-focused definitions.

Figure 2: The other mechanisms beyond physical proximity described here are quite interesting. Given that you are giving them focus in a figure, I would suggest discussing these in a bit more detail (rather than just one sentence) in the main text.

Figure 3: Can you summarize evidence for these different models of grammar.

Figure 5: There are MPRAs that are not episomal:
https://pubmed.ncbi.nlm.nih.gov/32641802/

Table 2: I suggest dropping this table or at least replacing the "mechanism" and "advantages" columns with specific examples of such methods. These models are too complex to accurately characterize in just a phrase or two. As a result, there are several misleading statements, for example every feature is not used in a decision tree. And many CNNs discussed here are not unsupervised.

**Reviewer 2**

This review, by Gabrielle Smith, Emily Wong, and colleagues, is broadly concerned with enhancers, ranging from what is known about where they are located and how they function, through their evolutionary properties, how they combine to enable robustness, experimental strategies for investigating their function, and the use of machine learning to predict their locations. The article is generally well written and clear, and it contains a good deal of useful information. I have a few minor quibbles with choices of topics, areas of emphasis, and references, but overall, I think it is solid review article that will be useful for readers new to the field.

I do think the review could be improved by addressing several limitations, as I detail below.

Major comments:

- The scope of the review is extremely broad. Whole review articles have been written to cover topics that are addressed here only in individual subsections—for example, regulatory evolution, applications of machine learning to regulatory genomics, and experimental methods for decoding sequence-function relationships. I would suggest making it clearer up front that this is a kind of "uber-review", spanning many different subfields, and as a result, the depth in each area is limited. Where appropriate, the authors should take care to cite recent narrower reviews on each of these more specific topics and refer the readers to them for additional depth and detail. Some examples close at hand (there are many others): https://www.annualreviews.org/doi/10.1146/annurev-genom-083118-014845, https://www.nature.com/articles/nrg3481; https://www.nature.com/articles/s41576-022-00532-2; https://www.nature.com/articles/s41576-021-00424-x; https://www.nature.com/articles/s41576-020-00311-x; https://www.nature.com/articles/nrg.2016.4; https://www.annualreviews.org/doi/10.1146/annurev-genom-122220-093818; https://www.annualreviews.org/doi/10.1146/annurev-genom-082509-141651

- At the same time, the review implicitly limits its scope in certain ways that could be made more clear to the reader. For example, while the title and abstract refer generally to enhancers, the review is primarily concerned with metazoans. Strikingly, there is no mention at all of plants, despite considerable interest in enhancers among plant biologists. I would suggest either being clearer in the title and abstract about the true scope of the article, or making some attempt to broaden it to cover all branches of life. For example, Fig 1 showing the proportion of DHS regions in human/mouse/fly genomes could easily be extended with such information from Arabidopsis or rice where DHS maps are readily available

Minor comments:

- The section entitled "Evolutionary trajectories of enhancers" (pp 5-7) repeatedly asserts that enhancers are not strongly conserved at the sequence level: e.g., "Most enhancers appear to evolve without strong sequence constraint" (p. 5, l. 13); "Given their fast evolutionary rate...." (p. 5, l. 41); "Despite the lack of detectable sequence conservation..." (p. 5, l 41). I found these claims to be somewhat overstated. It is true that enhancers, in general, are less constrained than protein-coding genes, many classes of noncoding RNAs, and promoters, but most do still exhibit detectable sequence constraint, at least across modest evolutionary distances (e.g,. within the mammals) and a subset are highly conserved (as the authors do note later on).

- There is quite a large literature now on the use of eRNAs to identify and characterize enhancers, and I felt it was given a rather cursory treatment here. The Lis and Yu labs at Cornell have a number of relevant papers (e.g., https://www.nature.com/articles/s41587-022-01211-7; https://www.nature.com/articles/s41588-018-0234-5; https://www.nature.com/articles/ng.3142; https://www.nature.com/articles/s41588-020-0686-2). The Danko lab has produced a widely used computational prediction method for enhancers that focuses specifically on this signal (https://www.nature.com/articles/nmeth.3329; https://genome.cshlp.org/content/29/2/293). Sandelin, Andersson and colleagues have also done extensive work in this area using CAGE data (e.g., https://www.nature.com/articles/nature12787). There are many other relevant works that could be mentioned.

- p. 7, l. 35-37. Suggest rewording the final sentence in this paragraph. A literal reading seems to suggest that such genetic variants will always or at least frequently become fixed, when in fact I think you mean to say that, if they do become fixed, it will be because of drift (most such mutations will not fix).

- p. 12, l. 15-43: This paragraph introduces a distinction between "classification" and "clustering", by which I think the authors mean supervised and unsupervised methods, etc. But supervised and unsupervised methods are more general in that they can extend beyond problems of assigning elements to discrete sets (e.g., as in regression). Later in the paragraph, the terms "supervised" and "unsupervised" are used but not explicitly connected to classification and clustering, which might leave a naive reader confused. I would suggest instead making the primary distinction between supervised and unsupervised methods and perhaps introducing classification vs. clustering as one example.

- Along the same lines, the authors somewhat gloss over what seems to me to be the most fundamental limitation of supervised ML methods for regulatory element identification—that, at least at present, labeled training data is generally quite incomplete and often heavily biased toward certain types of elements (e.g., ones in promoters, close to exon boundaries, or nearby well-studied genes; or, in the case of genome-wide multi-omic data, based on an inadequate surrogate for the regulatory functions of interest). Modern ML methods are extremely effective at prediction but in the end they are only as good as their training sets. To me, this is the main reason they do not yet dominate in regulatory element prediction the same way they do in, say, NLP or image labeling.

- P. 12, l. 30-35: The authors briefly mentioned cross-species model predictions here. They might consider addressing an interesting recent use of domain-adaptive methods for cross-species prediction (https://genome.cshlp.org/content/32/3/512.short), either in the immediate context, or later in the paragraph that discusses deep learning models.

- The authors might also consider expanding their discussion of interpretation (p. 14, l. 8-44) to allow that, while current methods such as DeepLIFT and DeepExplainer have made progress in interpreting the importance or relevance of individual sequences, it remains quite unclear how to extend NN methods to tackle the "decoding" of complex enhancer modules and grammars. This may be worth highlighting as a critical area for future work and a key for making such "black box" methods really useful.

- Table 2: The issue of interpretation is sufficiently important that it may be worthwhile to add a column devoted to it in Table 2. The goal would be to give readers a high-level overview of the tradeoffs in interpretability across different machine learning methods and perhaps point them toward techniques that can be used to extract biological insights from each type of model.

**Reviewer 3**

Enhancers are non-protein coding genomic DNA elements that control spatiotemporal gene
expression. However, while current methods in machine learning enable enhancer identification and the inference of the transcriptional impact of genetic variants with a high degree of accuracy, the biological understanding of key sequence features remains a significant challenge. To better understand enhancer activity, Dr. Wong and his collogues described the current challenges and the excited developments in high-throughput molecular methods and in silico methods in this manuscript, which provide a comprehensive mechanistic knowledge of the sequence's requirements, enhancer evolution, and their transcriptional networks for context-specific transcriptional regulation in mammals. The paper is generally well written and structured, and the figures in this manuscript are also appropriate, all the listed methods in the table are precisely cited. Overall, I am supportive of the manuscript.

**Authors Response**

**Point-by-point responses to the reviewers’ comments:**

***We thank the editor and all reviewers for their thoughtful comments and suggestions. We have prepared a point-by-point response below.***

***We have made changes to address all the points raised by the reviewers and have added a new section on current opportunities and challenges in the field as you suggested. Additionally, we have included more text to address current gaps in knowledge and aimed to provide more synthesis and perspective for readers with subject matter expertise. In the revised manuscript, we also made changes for readability and flow, which do not affect the content. We hope that our efforts will make the review more useful and informative for the readers.***

Reviewer reports:

Reviewer #1: This manuscript provides a review of much recent work on the prediction, validation, and evolution of enhancer sequences and their activity. There's a lot of ground to cover under this scope, and the review touches on most of the relevant literature; however, given the length, it is necessarily somewhat high-level.

I feel that this review would be useful for somone new to the field to help orient them to main themes and approaches. However, I do not think it provides sufficient synthesis, novel perspective, or provocative argument to be of much interest to experts. This is ok if an introduction was the goal, but I prefer reviews that have something for both the novice and expert. I would minimally suggest adding more concrete description of areas in need of future work, beyond the general "machine learning" and "high-throughput assays." What specifically within these areas are the opportunities? What are the challenges of integrating them and validating models?

***Thank you for the helpful suggestions. I have added a final section detailing opportunities and challenges. I tried to provide more synthesis to connect the findings of MPRA and ML methods to the more developmentally driven view of enhancers. I have also reworked the introduction, based on both reviewers’ suggestions to make the scope of the review clearer given the breadth of the topics covered.***

Here are my detailed comments and suggestions (page.line):

2.3 Typo: enhancers'

***Fixed***

2.6 This statement is unclear. Does this describe evolutionary sequence conservation or conservation of activity? I also feel that using the word "exception" suggests that enhancers are very rarely conserved, which is not true.

***Fixed***

2.24 This is incorrect. Enhancers can also occur in coding regions. See for example:

https://genome.cshlp.org/content/22/6/1059

***I have added this reference.***

2.30 The first paragraph of the Intro is very light on references. Perhaps citing a few relevant earlier reviews could help fill in the gaps.

***This is now done.***

2.41 I appreciated the detailed discussion of all the features that enhancers sometimes exhibit in this paragraph. However, I think it would be helpful to clarify at the start of the paragraph that these are neither all necessary or sufficient for enhancer activity. This become clear by the end of the paragraph, but was a bit confusing at the start.

***Yes, added (Intro 2nd paragraph)***

3.2 Can you clarify what exactly activity means here. How is it defined? Be a bit more explicit about the distinction being made.

***Yes, this has been included in above mentioned paragraph.***

3.24-28 While agree that a sequence-only approach would be great, it isn't totally clear to me that this is possible. Perhaps it isn't all specified in the sequence.

***Certainly epigenetic marks is a correlate of enhancer activity although the mechanisms of how they interplay with sequence and other epigenetic and transcriptional features are challenging to decipher. That said, multiple studies have shown that candidate enhancers can be classified, very accurately, in a cell-type-specific manner based on chromatin accessibility (e.g. Chen et al. 2022, Chen & Capra 2020, Kelley et al 2016). I have now clarified this in the introduction and noted that although it may not be possible to fully capture the complexity of enhancer activities using only a sequence-based ML approach, such a model can provide a solid foundation for understanding the limits of what can be learnt from the sequence.***

3.59 While it is interesting to mention transvection, I found its mention right at the start of the section on mechanisms without further comment a bit jarring. Perhaps discussing later after discussing the physical interaction model would be more clear.

***Agreed, I have changed this.***

4.10 "These features may not be universal" I agree! This could be the theme of studying enhancers!

***I have made this clearer.***

5.1 Typo: constraint

***Thanks, fixed.***

5.6 About the introduction of the different enhancer "grammar" models, I agree that this is necessary for context on the field. However, I think it would be helpful to be clear that these are not universal and there is unlikely to be a single answer. Enhancers likely exist along a spectrum between these extremes.

***Yes, I have made this clearer.***

5.30 I don't think "strategies" is the right word, it implies agency to the enhancers.

***Reworded.***

5.40 Does this mean confirmed functional activity conservation? There are many functionally (and sequence) conserved human-zebrafish enhancers that probably haven't been tested in more diverged animals. I wonder how many of these at least have evidence of sequence conservation across other animals?

***No, it doesn’t mean that activity is confirmed – it merely suggests that there is an enhancer present which must then be validated. There are many studies that have compared fish and mammals using transgenic assays to assess activity at specific loci (McGaughey et al 2008. Metrics of sequence constraint overlook regulatory sequences in an exhaustive analysis at phox2b. Genome Res. 18: 252–60, Fisher et al. 2006. Conservation of RET regulatory function from human to zebrafish without sequence similarity. Science 312: 276–79). Pennacchio e al. 2006. showed human-pufferfish conservation could moderately predict enhancer function. ~29% of the enhancers they tested based on conservation showed activity (caveat: less than 10 were tested). Across all metazoans – e.g. between human and sea sponge it is not possible to detect any candidate enhancers conserved by general sequence metrics alone– which is why the syntenic regions are useful to indicate functional elements that may be conserved.***

6.35 In this paragraph, I was confused about how the discussion of somatic variation related to differences in germline variation. Can this be clarified?

***I wanted to express that not only germline variation contribute to enhancer repertoires because somatic mutations can also play a role. For example, biological replicates of enhancers profiled in a particular tissue may be more heterogeneous in tissues with higher cell turnover. I think there is not enough context to add this in there. I’ve removed it to avoid confusion.***

7.35 I find the definition and discussion of robustness to be unclear. For example, why would this necessarily imply that variants evovle neutrally. For example, shadow enhancers are more conserved than expected. I suggest clearly defining the meaning of robustness in this discussion at the start. I also suggest citing and referring to this recent review on enhancer robustness:

https://www.nature.com/articles/s41576-020-00311-x

***I have added this reference into the text. I’ve also added in a review on biological robustness (Kitano 2004 NRG). Not all variants would evolve more neutrally – only those that are robust. I have clarified this. [“Enhancer sequences that are robust would be expected to possess a higher proportion of genetic sequences that do not affect fitness. These variants would be expected to evolve more neutrally.”]***

7.46 Can this estimate of the time of emergence be explained? What is the starting point?

***Yes, I have provided more details in the text. This was simulated by evolving 30 regions from genomic background in Drosophila and compared to known regulatory sequences involved in anterior-posterior axis specification at the blastoderm stage. Each sequence was 500-2000 bp long. Evolution was model via a Wright-Fisher model. No recombination is considered but selection is included. They used a thermodynamic model to relate the sequence to expression. They modelled both binding site gain and loss across the entire sequence.***

10.8 Typo: provide

***Thanks.***

10.17/55 What is meant by "diseased"?

***Thanks, this is an enhancer that contains a putative disease-causing variant. I have added the explanation.***

11.2 I feel that more discussion is needed about the lack of agreement/replication and other challenges in interpreting the results of different MPRAs. Reference 153 gets at this, but this challenge isn't described in the text.

***I have added another sentence to explain the challenges.***

11.26 It would be helpful to clarify from the start that these ML models are often not trained on validated enhancers but regions with signatures associated with enhancers.

***I have reworked the introduction and the final section on challenges and opportunities to make sure that this is now highlighted. This is also mentioned throughout the review where appropriate.***

11.42 This paragraph is lacking citations throughout. Cite relevant reviews or primary lit.

***Thanks, I have reworded and added references.***

11.48 I disagree with the characterization of ML models into neural network and non-neural network. The non-neural network methods are quite diverse and distinct.

***Thank you for the point. I have reworded to make clear that the non-neural network algorithms are discrete types of machine learning methods. This was a general grouping only because the field now dominated by neural network-based methods and was not mean to imply that all non-neural network methods were similar.***

13.46 I'd suggest also citing the Enformer method, since it also predicts many markers of enhancer activity:

https://www.nature.com/articles/s41592-021-01252-x

***Thanks, this reference is added but referenced earlier with other reference related to gene expression prediction.***

13.50 Reference for this?

**Added.**

Figure 1: Given the dependence of these estimates on ChromHMM, it would be helpful to explain this method.

***I have added a simple explanation in the figure legend. [‘Human epigenomics data from the Roadmap Epigenomics [213] project, comprising of 98 cell and tissue types, were grouped to functional categories using ChromHMM [214] based on their combination of histone modification profiles’]***

Figure 1: Also, how are these fractions sensitive to the very different numberes of cellular contexts included?

***Many enhancers are cell type specific e.g. only 28% of the enhancers from 6 human cell lines (K562, Gm12878, Hepg2, H1hesc, Huvec, Helas3) are found in more than one line. This is why we focused on human and mouse where many epigenomic datasets are available - 55 cell types in mouse and 98 in humans. In the consortium datasets, multiple replicates are used so these should be somewhat robust and the data are processed in a standardized manner, thus reducing variability. In flies we have data from whole animals during development and so this should cover the major cell types. Of note, we have added in Epigeneomics Roadmap data which have increased the proportion of candidate enhancer regions but as this dataset is the most comprehensive epigenetic dataset available. We feel that it is important to***

Figure 1: Given the debates surrounding the ENCODE-style definitions of function, it would be worth discussing this debate slightly and mentioning conservation-focused definitions.

***I have emphasized and the reworded to make it clear that the encode derived enhancers are candidate enhancers and do not reflect enhancer activity. I have also expanded on the conservation-focused definitions and added this in an earlier section.***

Figure 2: The other mechanisms beyond physical proximity described here are quite interesting. Given that you are giving them focus in a figure, I would suggest discussing these in a bit more detail (rather than just one sentence) in the main text.

***We have expanded on this in the main text. [Phase separated condensates, a mechanism of biochemical compartmentalisation, may enable the action of super enhancers given parallels between the molecular cooperativity in cellular body formation and the assembly of regulatory factors at high density during super enhancer activation [61,62]. A hypothetical model of communication via diffusion, described as TF activity gradients (TAG), has been proposed to regulate enhancer-promoter communication via short-distance diffusion of acetylated TFs [63]. This model eliminates the need for physical contact between enhancer and promoter and provides a possible explanation for observations of proximity, but not contact, between some active enhancers and promoters. Another possible mode of enhancer action could involve the transcription of enhancers into eRNAs, which have been implicated in transcriptional regulation via interaction with NELF, stimulating Pol II pause release and transcriptional elongation and the stimulation of Pol II pause release through eRNAs [64].]***

Figure 3: Can you summarize evidence for these different models of grammar.

***As there are much more comprehensive reviews on this topic and this is challenging to do in the figure, I have added in references to relevant reviews in the main text and in the figure legend for interested readers (Long et al. Cell 2016, Jindal et al Dev Cell 2021)***

Figure 5: There are MPRAs that are not episomal:

https://pubmed.ncbi.nlm.nih.gov/32641802/

***Thanks, we had not meant to imply there are only episomal MPRAs as randomly integrated transgenes are also listed. We have made this clearer in the legend.***

Table 2: I suggest dropping this table or at least replacing the "mechanism" and "advantages" columns with specific examples of such methods. These models are too complex to accurately characterize in just a phrase or two. As a result, there are several misleading statements, for example every feature is not used in a decision tree. And many CNNs discussed here are not unsupervised.

***We have reworded and have added an Interpretations column in addressing Reviewer 2’s comment.***

Reviewer #2: This review, by Gabrielle Smith, Emily Wong, and colleagues, is broadly concerned with enhancers, ranging from what is known about where they are located and how they function, through their evolutionary properties, how they combine to enable robustness, experimental strategies for investigating their function, and the use of machine learning to predict their locations. The article is generally well written and clear, and it contains a good deal of useful information. I have a few minor quibbles with choices of topics, areas of emphasis, and references, but overall, I think it is solid review article that will be useful for readers new to the field.

I do think the review could be improved by addressing several limitations, as I detail below.

Major comments:

- The scope of the review is extremely broad. Whole review articles have been written to cover topics that are addressed here only in individual subsections—for example, regulatory evolution, applications of machine learning to regulatory genomics, and experimental methods for decoding sequence-function relationships. I would suggest making it clearer up front that this is a kind of "uber-review", spanning many different subfields, and as a result, the depth in each area is limited. Where appropriate, the authors should take care to cite recent narrower reviews on each of these more specific topics and refer the readers to them for additional depth and detail. Some examples close at hand (there are many others): https://www.annualreviews.org/doi/10.1146/annurev-genom-083118-014845, https://www.nature.com/articles/nrg3481;https://www.nature.com/articles/s41576-022-00532-2;https://www.nature.com/articles/s41576-021-00424-x;

https://www.nature.com/articles/s41576-020-00311-x;https://www.nature.com/articles/nrg.2016.4;https://www.annualreviews.org/doi/10.1146/annurev-genom-122220-093818; https://www.annualreviews.org/doi/10.1146/annurev-genom-082509-141651

***Many thanks, we have taken on board the suggestion and added these references in their most appropriate places.***

- At the same time, the review implicitly limits its scope in certain ways that could be made more clear to the reader. For example, while the title and abstract refer generally to enhancers, the review is primarily concerned with metazoans. Strikingly, there is no mention at all of plants, despite considerable interest in enhancers among plant biologists. I would suggest either being clearer in the title and abstract about the true scope of the article, or making some attempt to broaden it to cover all branches of life. For example, Fig 1 showing the proportion of DHS regions in human/mouse/fly genomes could easily be extended with such information from Arabidopsis or rice where DHS maps are readily available

***Thank you. I have included a couple of sentences in the introduction on plant enhancers (which are relevant and interesting yet little studied) with a few key references. We mention the focus is on metazoans. [‘Although enhancer elements are also found in plants [28], we have focused on metazoan enhancers as they are have been most extensively studied. Enhancers have been identified in rice using massively parallel reporter assay (MPRA) [29], and chromatin accessibility maps are available for multiple cell types in Arabidopsis and rice [30,31]’].***

***Even though the histone marks for enhancers appear different in some plants compared to metaoans, the strategies for analyses can be relevant to readers in that field. Regarding adding to Fig 1 - it is difficult to make a fair comparison in Figure 1 with the currently limited data available in plants. With human mouse and fly there has been a lot of chromatin accessible/histone mark data generated across many cell types at multiple time points so the data for these species are a lot more representative.***

Minor comments:

- The section entitled "Evolutionary trajectories of enhancers" (pp 5-7) repeatedly asserts that enhancers are not strongly conserved at the sequence level: e.g., "Most enhancers appear to evolve without strong sequence constraint" (p. 5, l. 13); "Given their fast evolutionary rate...." (p. 5, l. 41); "Despite the lack of detectable sequence conservation..." (p. 5, l 41). I found these claims to be somewhat overstated. It is true that enhancers, in general, are less constrained than protein-coding genes, many classes of noncoding RNAs, and promoters, but most do still exhibit detectable sequence constraint, at least across modest evolutionary distances (e.g,. within the mammals) and a subset are highly conserved (as the authors do note later on).

***I have reworded to make this more precise and to avoid overstatements and added appropriate references where needed.***

- There is quite a large literature now on the use of eRNAs to identify and characterize enhancers, and I felt it was given a rather cursory treatment here. The Lis and Yu labs at Cornell have a number of relevant papers (e.g., https://www.nature.com/articles/s41587-022-01211-7;https://www.nature.com/articles/s41588-018-0234-5;https://www.nature.com/articles/ng.3142;https://www.nature.com/articles/s41588-020-0686-2). The Danko lab has produced a widely used computational prediction method for enhancers that focuses specifically on this signal (https://www.nature.com/articles/nmeth.3329;https://genome.cshlp.org/content/29/2/293). Sandelin, Andersson and colleagues have also done extensive work in this area using CAGE data (e.g., https://www.nature.com/articles/nature12787). There are many other relevant works that could be mentioned.

***Many thanks, I have added in these key references.***

- p. 7, l. 35-37. Suggest rewording the final sentence in this paragraph. A literal reading seems to suggest that such genetic variants will always or at least frequently become fixed, when in fact I think you mean to say that, if they do become fixed, it will be because of drift (most such mutations will not fix).

**I have reworded this section.**

- p. 12, l. 15-43: This paragraph introduces a distinction between "classification" and "clustering", by which I think the authors mean supervised and unsupervised methods, etc. But supervised and unsupervised methods are more general in that they can extend beyond problems of assigning elements to discrete sets (e.g., as in regression). Later in the paragraph, the terms "supervised" and "unsupervised" are used but not explicitly connected to classification and clustering, which might leave a naive reader confused. I would suggest instead making the primary distinction between supervised and unsupervised methods and perhaps introducing classification vs. clustering as one example.

***Thanks for the point, I have changed this to make the distinction between supervised and unsupervised methods.***

- Along the same lines, the authors somewhat gloss over what seems to me to be the most fundamental limitation of supervised ML methods for regulatory element identification—that, at least at present, labeled training data is generally quite incomplete and often heavily biased toward certain types of elements (e.g., ones in promoters, close to exon boundaries, or nearby well-studied genes; or, in the case of genome-wide multi-omic data, based on an inadequate surrogate for the regulatory functions of interest). Modern ML methods are extremely effective at prediction but in the end they are only as good as their training sets. To me, this is the main reason they do not yet dominate in regulatory element prediction the same way they do in, say, NLP or image labeling.

***Yes I agree with the bias. E.g. the validated human enhancers in VISTA are also always conserved in mouse. And human cell lines enhancer data is highly abundant but of limited use for understanding phenotype. I have highlighted that validated enhancers (with activity data) are rare and validating activity in the correct cell type/time point a major challenge.***

- P. 12, l. 30-35: The authors briefly mentioned cross-species model predictions here. They might consider addressing an interesting recent use of domain-adaptive methods for cross-species prediction (https://genome.cshlp.org/content/32/3/512.short), either in the immediate context, or later in the paragraph that discusses deep learning models.

***Thanks, I have added this reference and added other references which also make cross-species predictions in the first paragraph of the ML section.***

- The authors might also consider expanding their discussion of interpretation (p. 14, l. 8-44) to allow that, while current methods such as DeepLIFT and DeepExplainer have made progress in interpreting the importance or relevance of individual sequences, it remains quite unclear how to extend NN methods to tackle the "decoding" of complex enhancer modules and grammars. This may be worth highlighting as a critical area for future work and a key for making such "black box" methods really useful.

***Thanks, I have added to the section on future directions. The integration of machine learning with experimental validation will be key to fully realizing the potential of these approaches to decipher the intricate relationship between enhancer sequences and activity. Also, another area is how to use these tools to discover new biology beyond confirming what we know (ie key characterized motifs). I have added that NLP may be useful for more interpretable models and proteomics can be used to validate kmers to gain novel insights.***

- Table 2: The issue of interpretation is sufficiently important that it may be worthwhile to add a column devoted to it in Table 2. The goal would be to give readers a high-level overview of the tradeoffs in interpretability across different machine learning methods and perhaps point them toward techniques that can be used to extract biological insights from each type of model.

***Thanks, we have added an Interpretations column with references as suggested.***

**Second round of review**

**Reviewer 1**

I thank the authors for addressing many of my comments and suggestions. I believe that the manuscript has improved and will provide valuable context for readers new to the field.

One suggestion unrelated to the manuscript: in the future when responding to reviewer comments, please use a format that makes it easy to distinguish reviewer comment from your response.

**Reviewer 2**

I think the authors have done a reasonably good job in responding to the reviewers' comments. There are many areas of the review that could still be expanded or strengthened, but the topic is broad, and I understand that compromises have to be made. Overall, the review reads well and will be useful to the community.
